# Fundamental energy cost of finite-time parallelizable computing

**DOI:** 10.1038/s41467-023-36020-2

**Published:** 2023-01-27

**Authors:** Michael Konopik, Till Korten, Eric Lutz, Heiner Linke

**Affiliations:** 1 NanoLund and Solid State Physics, Lund University, S-22100 Lund, Sweden; 2 Institute for Theoretical Physics I, University of Stuttgart, D-70550 Stuttgart, Germany; 3 B CUBE - Center for Molecular Bioengineering and Cluster of Excellence Physics of Life, Technische Universität Dresden, D-01307 Dresden, Germany

**Keywords:** Information theory and computation, Thermodynamics

## Abstract

The fundamental energy cost of irreversible computing is given by the Landauer bound of $kT\ln 2$/bit, where *k* is the Boltzmann constant and *T* is the temperature in Kelvin. However, this limit is only achievable for infinite-time processes. We here determine the fundamental energy cost of finite-time parallelizable computing within the framework of nonequilibrium thermodynamics. We apply these results to quantify the energetic advantage of parallel computing over serial computing. We find that the energy cost per operation of a parallel computer can be kept close to the Landauer limit even for large problem sizes, whereas that of a serial computer fundamentally diverges. We analyze, in particular, the effects of different degrees of parallelization and amounts of overhead, as well as the influence of non-ideal electronic hardware. We further discuss their implications in the context of current technology. Our findings provide a physical basis for the design of energy-efficient computers.

## Abstract (web summary)

Based on fundamental thermodynamics, traditional electronic computers, which operate serially, require more energy per computation the faster they operate. Here, the authors show that the energy cost per operation of a parallel computer can be kept very small.

## Introduction

There is wide agreement that Moore’s law regarding the exponential growth of the number of components in integrated circuits^1^ is coming to an end^2,3^. One of the main physical reasons that prevents further miniaturization is unavoidable heat generation^2,3^. A much-improved energy efficiency of computing is therefore a key requirement for any ‘More-than-Moore’ technology^4^. The fundamental limits to the work cost and the heat dissipation of computing are given by the Landauer bound of $kT\ln 2$ per logically irreversible bit operation^5^, where *k* is the Boltzmann constant and *T* the temperature. The existence of such a lower limit has been recently established in a number of classical^6–11^ and quantum^12,13^ experiments. However, the Landauer bound is only asymptotically reachable for quasistatic processes^14,15^. In reality, however, all computing tasks take place in finite time^16–24^, and the energy cost per operation necessarily increases with operation frequency.

Empirically, the rapid growth in power consumption with increasing processor frequency has triggered, in the past two decades, a switch to increased parallelization in order to achieve performance gains^25,26^. Parallel processors have by now become mainstream^27^, however, their finite-time energy consumption limits have not been investigated so far. We here seek the fundamental minimal energy cost of finite-time parallelizable computing using the tools of nonequilibrium thermodynamics^28^. Our aim is to complement discussions of ultimate limits, which, while essential, possess only little practical relevance^29^ or do not address the fundamental advantages of parallel computing^30^, and of more applied considerations^31,32^, with only restricted generality. A key insight of our study is that, when a given problem is to be solved in finite time, the energy cost per operation of a parallel computer can be kept close to the Landauer limit even for large problem sizes, whereas that of a serial computer fundamentally diverges. We further analyze how this result is affected by different degrees of parallelization and various amounts of overhead operations^27^, as well as the effect of leakage currents and provisioning^33–36^. We also consider the case of reversible computing^37,38^. We finally place our results quantitatively into the context of existing and emerging technologies^39–43^.

**Fig. 1:**
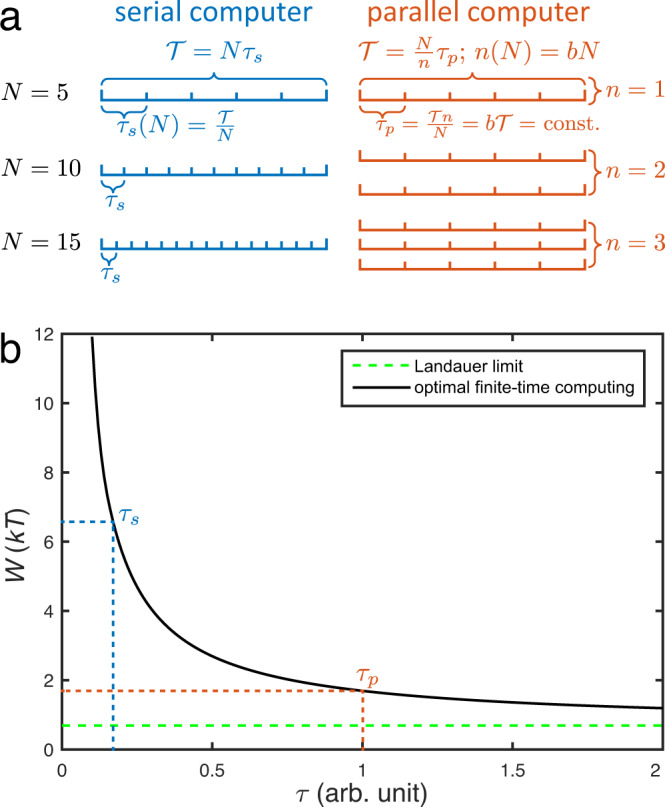
Assumptions for ideal serial and parallel computers. **a** Schematic illustration of the three main assumptions: (i) The total time ${{{{{{{\mathcal{T}}}}}}}}$ available to solve a given problem requiring *N* irreversible computing operations is limited; (ii) an ideal serial computer (left, blue) reduces the time per operation *τ*_s_ with increasing problem size *N*; (iii) an ideal parallel computer (right, red) is able to increase the number of processors *n* proportional to the size *N* in order to keep the time per operation *τ*_p_ constant. **b** Optimal 1/*τ* behavior of the energy consumption of a single algorithmic operation of duration *τ*, and the effects that the serial (blue) or parallel (red) computing strategies have on the energetic cost of computation.

We base our analysis on the following assumptions (Fig. 1): (i) Computing problems with a (variable) number *N* of logically irreversible operations should be solved in (constant) finite time ${{{{{{{\mathcal{T}}}}}}}}$. In order to stay within this time limit, (ii) an ideal serial computing strategy has to adapt dynamically its processing frequency (time per operation *τ*_s_; Fig. 1a left), whereas (iii) an ideal parallel computing strategy is able to adapt the number *n* of processors, keeping constant its processing frequency (time per operation *τ*_p_; Fig. 1a right). These assumptions are well justified. Assumption (i): While the available time is not exactly fixed, there is usually a limit on how long calculations can be run^27,44^. Assumptions (ii) and (iii) may be viewed as a minimal model of processors that are implemented in modern technology. A single CPU core indeed behaves similarly to our idealized serial computer by adapting its frequency to the workload using dynamic frequency and voltage scaling^31,32^. On the other hand, a multi-core CPU behaves like our idealized parallel computer by deactivating unused cores using deep-sleep states^45^.

## Results

### Nonequilibrium Landauer bound

Let us first consider a single algorithmic computation of duration *τ*. Because it occurs in finite time, such a nonequilibrium process is necessarily accompanied by the dissipation of an amount of work *W*_dis_ into the environment^28^. The energetic cost of a finite-time, logically irreversible bit operation may hence be written as a generalized Landauer bound,

1 $$W(\tau )=kT\ln 2+{W}_{{{{{{{{\rm{dis}}}}}}}}}(\tau ),$$

where *W*_dis_/*T* ≥ 0 is the nonequilibrium entropy produced during the process^28^. Equation (1) reduces to the usual Landauer limit for quasistatic computation, indicating that more work per operation is required for fast operations and, in turn, more heat is dissipated. The equilibrium contribution $kT\ln 2$ is obtained for fully mixed (that is, unbiased) memory states^15^.

Since we are interested in the fundamental energy bound, we focus on optimal protocols with minimal entropy production^16–19,22,24^. In that case, *W*_dis_ = *a*/*τ*, both for slow and fast bit operations^16–19,22,24^, where *a* is an energy efficiency constant that depends on the system (Fig. 1). In particular, the optimal 1/*τ* scaling has been shown to hold generically for any hardware implementation, for any time region (that is, slow, moderate and fast driving), for systems that fulfill detailed balance^22^. It has also been demonstrated to apply at all times to overdamped dynamics in the absence of detailed balance^16^, which is the case for realistic memories that store information in a nonequilibrium steady state. Such behavior has been observed experimentally close to equilibrium in overdamped^6–8^ and underdamped^21^ systems; it further appears for transitions between metastable states^46,47^. It is worth noting that the $kT\ln 2$ limit is valid for any (biased) statistics of the input memory state, when driving protocols are designed to be thermodynamically optimal for the fully mixed state^48^. This remark may be extended to ‘modularity dissipation’ which occurs when inputs to various computational units (an issue pertinent for parallelization) contain correlations^49–51^: If the computational units are designed to be thermodynamically optimal for uniform input, then $kT\ln 2$ will be payed for each bit operation regardless of the true input statistics^48^.

In view of Eq. (1), the total work cost associated with the solution of a computing problem that requires *N* bit operations within the finite time ${{{{{{{\mathcal{T}}}}}}}}$ is given by,

2 $${W}_{{{{{{{{\rm{tot}}}}}}}}}(N,\tau )=NW(\tau )=NkT\ln 2+N{W}_{{{{{{{{\rm{dis}}}}}}}}}(\tau ),$$

where $\tau=\tau ({{{{{{{\mathcal{T}}}}}}}})$ is in general a function of ${{{{{{{\mathcal{T}}}}}}}}$. The scaling of the dissipative term with the problem size *N* depends on the type of computing considered. It may be concretely determined for the two idealized computer models introduced above: (i) for an ideal serial computer, the available time per operation decreases with the problem size as ${\tau }_{{{{{{{{\rm{s}}}}}}}}}={{{{{{{\mathcal{T}}}}}}}}/N=1/{f}_{{{{{{{{\rm{op}}}}}}}}}$ (Fig. 1a left), whereas (ii) for an ideal parallel computer that solves the problem with a number of processors *n*(*N*) = *bN* (with *b* ∈ (0, 1]) that scales linearly with *N*, the time per operation stays constant, ${\tau }_{{{{{{{{\rm{p}}}}}}}}}=n{{{{{{{\mathcal{T}}}}}}}}/N=b{{{{{{{\mathcal{T}}}}}}}}$ (Fig. 1a right). The quantity *f*_op_ can be interpreted as the operation frequency of the serial processor, whereas 1/*b* determines the number of operations performed by each processor; in the following, we set *b* = 1 (the effect of different values of *b* is discussed in the Supplementary Information, Sec. S3). The fundamental total energy cost per operation for the serial implementation, therefore, scales with the problem size as,

3 $$\frac{{W}_{{{{{{{{\rm{tot}}}}}}}}}^{{{{{{{{\rm{ser}}}}}}}}}(N,{{{{{{{\mathcal{T}}}}}}}})}{N}=kT\ln 2+\frac{a}{{{{{{{{\mathcal{T}}}}}}}}}N=kT\ln 2+a{f}_{{{{{{{{\rm{op}}}}}}}}}.$$

The corresponding scaling for the parallel implementation reads,

4 $$\frac{{W}_{{{{{{{{\rm{tot}}}}}}}}}^{{{{{{{{\rm{par}}}}}}}}}(N,{{{{{{{\mathcal{T}}}}}}}})}{N}=kT\ln 2+\frac{a}{b{{{{{{{\mathcal{T}}}}}}}}}.$$

**Fig. 2:**
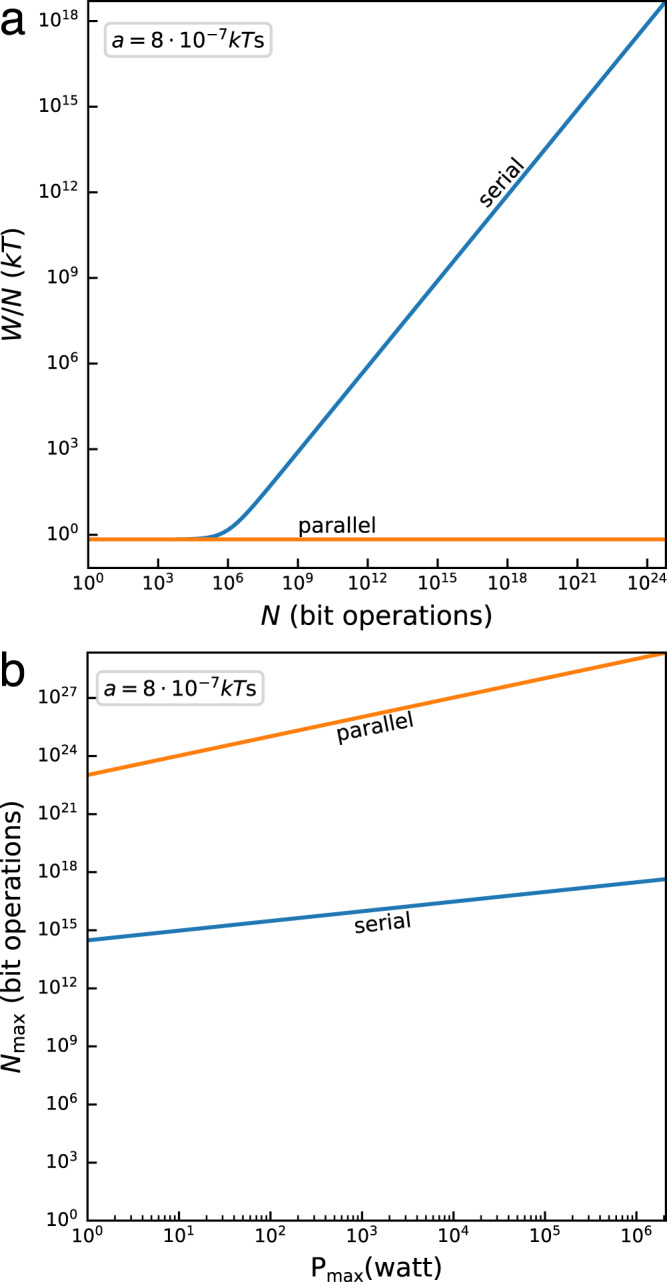
Finite-time Landauer bound for ideal serial and parallel computers. **a** Energy consumption per operation, *W*/*N*, for solving a fully parallelizable problem of size *N* by an ideal serial, Eq. (3) (blue), and parallel, Eq. (4) (orange), computer. The energetic cost diverges with *N* for an ideal serial computer and remains constant for an ideal parallel computer. **b** Maximal number of bit operations ${N}_{\max }^{{{{{{{{\rm{ser}}}}}}}}}\,{N}_{\max }^{{{{{{{{\rm{par}}}}}}}}}$ that can be performed by an ideal serial, Eq. (5) (blue), and parallel, Eq. (6) (orange), computer in the finite time ${{{{{{{\mathcal{T}}}}}}}}=1$ s within a given power budget ${P}_{\max }$. Parameters are *T* = 1 K, *b* = 1 and *a* = 8⋅10^−7^*kT*s.

Equations (3) and (4) highlight an important, fundamental difference between serial and parallel computing: whereas the energy cost per operation for a serial computer necessarily increases at least linearly with *N*, the energy cost per operation for an ideal parallel computer is independent of *N* (Fig. 2a); it depends only on the two constants *a* and *b* as well as the chosen ${{{{{{{\mathcal{T}}}}}}}}$. If the computation task permits to choose a large ${{{{{{{\mathcal{T}}}}}}}}$, then the finite-time energy cost per operation for the parallel computer is bounded only by the Landauer limit, even for very large *N*. Equations (3) and (4) further imply that for a computer with a maximum power budget ${P}_{\max }={W}_{\max }/{{{{{{{\mathcal{T}}}}}}}}$, the maximal problem size ${N}_{\max }$ that can be solved within the (fixed) time limit ${{{{{{{\mathcal{T}}}}}}}}$ is proportional to the square root of the power $\sqrt{{P}_{\max }}$ for a serial implementation

5 $${N}_{\max }^{{{{{{{{\rm{ser}}}}}}}}}({P}_{\max },{{{{{{{\mathcal{T}}}}}}}})=\frac{\sqrt{4a{P}_{\max }+{(kT\ln 2)}^{2}}}{2(a/{{{{{{{\mathcal{T}}}}}}}})}-\frac{kT\ln 2}{2(a/{{{{{{{\mathcal{T}}}}}}}})},$$

whereas it is directly proportional to the power ${P}_{\max }$ for a parallel implementation

6 $${N}_{\max }^{{{{{{{{\rm{par}}}}}}}}}({P}_{\max },{{{{{{{\mathcal{T}}}}}}}})=\frac{{P}_{\max }{{{{{{{\mathcal{T}}}}}}}}}{kT\ln 2+(a/b{{{{{{{\mathcal{T}}}}}}}})}.$$

An ideal parallel computer can therefore, in principle, solve quadratically bigger problems within the same time and energy constraints as an ideal serial computer (Fig. 2b). Within the power budget range of 1 W–400 MW shown in Fig. 2b, the ideal parallel computer solves problems that are 7 to 12 orders of magnitude larger than the problems solved by an ideal serial computer under the same power constraints. We note that the constants *a* and *b* depend on the topology of the circuit and may be determined empirically. While different constants would lead to quantitatively different results, the quadratic advantage of the ideal parallel computer is fundamental and independent of the specific circuit used.

**Fig. 3:**
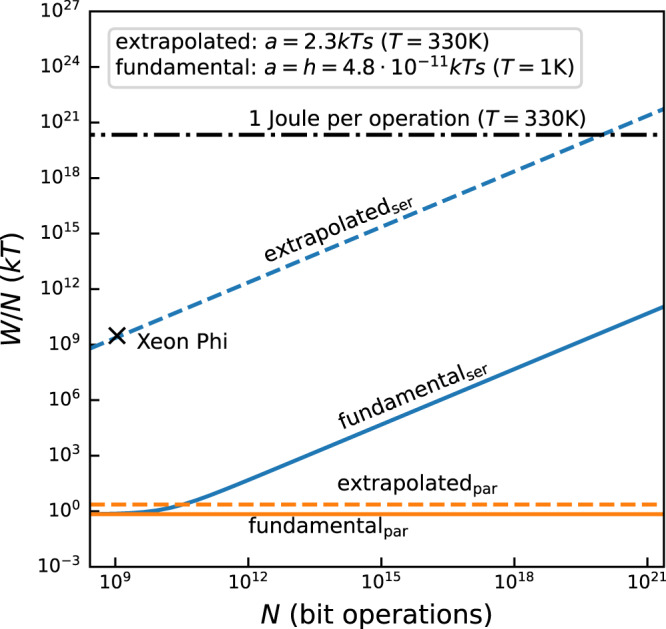
Fundamental limit and extrapolated energy cost per operation for ideal serial and parallel computers. Fundamental limits obtained for *a* = *h* (Planck constant; *T* = 1*K*) (solid lines) and extrapolated energy cost corresponding to *a* = 2.3*kT*s (Xeon Phi; *T* = 330 *K*) (dashed lines) for ideal serial, Eq. (3) (blue), and parallel, Eq. (4) (orange), computers. The measured value for a Xeon Phi processor is represented by a black X. For reference, an energy cost of 1 J/operation is shown as a dash-dotted line. Parameters are ${{{{{{{\mathcal{T}}}}}}}}=1$ s and *b* = 1.

To understand the practical importance of the finite-time energy cost, quantitative values for *a* are required. A state-of-the-art general purpose processor that is highly parallel, runs at a relatively low clock rate (60 cores à 4 threads at *f*_op_ = 1.09 GHz), and has been thoroughly analyzed for its energy consumption is the Intel Xeon Phi: it consumes 4.5⋅10^−10^ J/32 bit operation or *a* ⋅ *f*_op_ = 1.4⋅10^−11^ J/operation^52^ (note that this value accounts only for computation operations and ignores more costly transfers to and from memory). This allows us to obtain *a* = 1.0⋅10^−20^Js ≈ 2.3 *kT*s (*T* = 330 K, the typical operating temperature of a CPU under load) as an estimate for electronic computers (Fig. 3, dashed lines). This implies that the finite-time energy cost of an electronic computer exceeds the (quasistatic) Landauer limit already at a few Hertz of operation frequency.

Fundamentally, one may argue that the lowest possible value for *a* is quantum mechanically given by Planck’s constant, *h* = 6.6 ⋅ 10^−34^ Js ≈ 4.8⋅10^−11^ *kT*s (at *T* = 1*K*)^30^, 13 orders of magnitude lower than the above value for current electronic computers (Fig. 3, solid lines). However, to our knowledge, no physical system has been proposed that would reach such a small value for *a*. In recent experimental studies of the thermodynamics of finite-time operations, much higher values have been found. The lowest measured value known to us is *a* = 1.1⋅10^−29^ Js, reported for memory operations using molecular nanomagnets^13^, corresponding to *a* = 8⋅10^−7^ *kT*s at the operation temperature of *T* ≈ 1 K. For comparison, for experiments with optical traps, *a* ≈ 2 *kT*s = 8⋅10^−22^ Js at room temperature^19^.

Based on these insights, it is illustrative to compare the fundamental energy cost of finite-time computing as a function of problem size *N* for fully serial and fully parallel computers (Fig. 3). For a serial, electronic computer (blue dashed line) with representative *a* = 2.3 *kT*s (*T* = 330 K, the typical operating temperature of a CPU under load), the finite-time energy cost is dominated by the term $aN/{{{{{{{\mathcal{T}}}}}}}}$ in Eq. (3). A further increase in *N* (corresponding to an increase in operation frequency ${f}_{{{{{{{{\rm{op}}}}}}}}}=N/{{{{{{{\mathcal{T}}}}}}}}$ of a serial computer beyond the currently typical *f*_op_ ≈ 1 GHz) thus leads to a continued increase in energy dissipation per operation. Given that thermal management is already now the limiting factor in processor design, this is not an option unless *a* can be lowered, for example through transistor and circuit design. If, on the other hand, the quantum mechanical limit of *a* ≈ *h* were achievable for a serial computer, then the term $aN/{{{{{{{\mathcal{T}}}}}}}}$ in Eq. (3) would become noticeable, compared to the frequency-independent Landauer limit, *as soon as N* exceeds 10^10^ operations, corresponding to *f*_op_ ≈ *O*(10 GHz) (blue line). By contrast, a fully (ideal) parallel computer does not increase its energy cost per operation (orange lines). For *a* = 2.3 *kT*s (Xeon Phi) and *τ*_p_ = 1 s, the extrapolated energy cost per operation (orange dashed line) is only about one order of magnitude larger than the fundamental Landauer bound (orange solid line).

### Partial parallelization

**Fig. 4:**
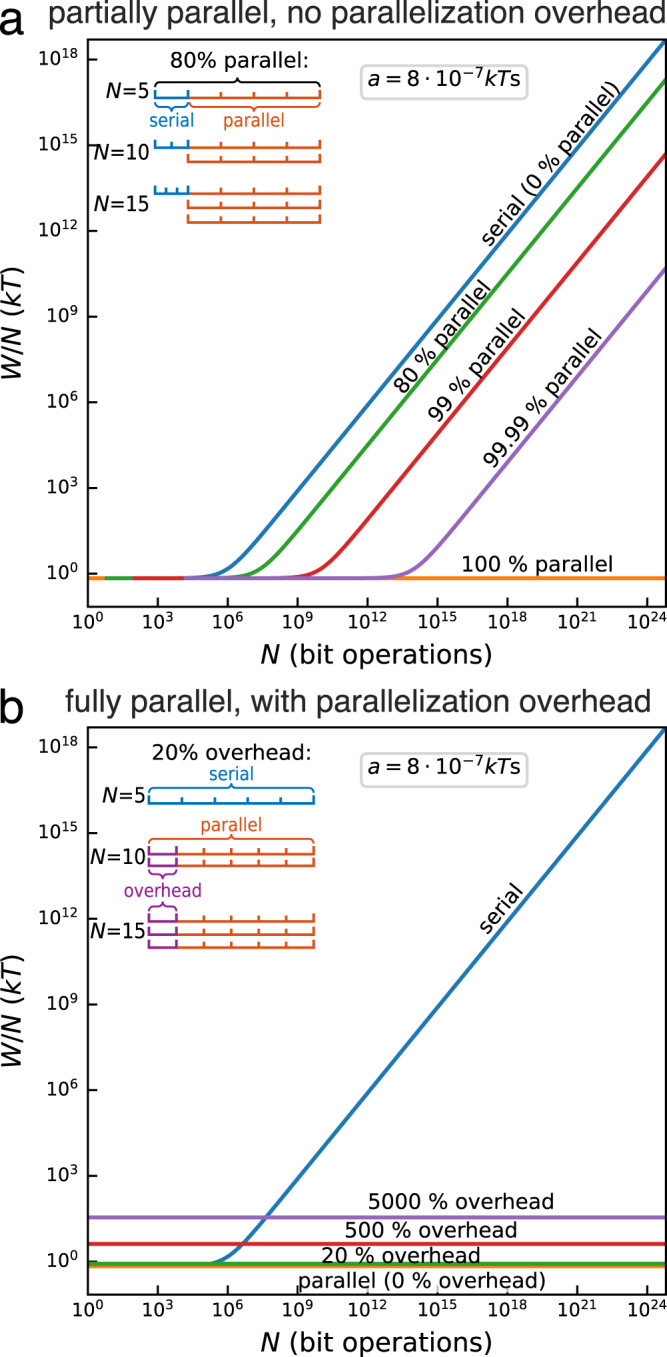
Effects of not ideally parallelizable problems. **a** Energy cost per operation ${W}_{{{{{{{{\rm{tot}}}}}}}}}^{{{{{{{{\rm{com}}}}}}}}}/N$ for a partially parallelizable algorithm that has no overhead, Eq. (7). **b** Energy cost per operation ${W}_{{{{{{{{\rm{tot}}}}}}}}}^{{{{{{{{\rm{ove}}}}}}}}}/N$ for a fully parallel algorithm with linear overhead *N*_ove_(*n*) = *cn*, Eq. (9), and *c* = 0 − 5000%. Same parameters as in Fig. 2.

Real-world algorithms may not be completely parallelizable. Therefore, the ideal estimates, Eqs. (3) and (4), need to be refined. The impact of non-parallelizable parts of an algorithm on the speedup of parallel computing is commonly described by Amdahl’s law^27^. According to Amdahl^53^, the time of the initial serial realization ${{{{{{{\mathcal{T}}}}}}}}$ can be split into two contributions, a purely serial part *s*, that cannot be done by more than one processor at a time, and a parallel part *p* that can, ideally, be split equally among all the used *n* processors (Fig. 4a inset). We evaluate the energetic consequences of such a splitting for our ideal computers as follows: We assume that a given problem of size *N* is comprised of a serial and parallel part, *N* = *N*_s_ + *N*_p_ = *sN* + *pN*. The total computation time is given by the sum of these two parts, ${{{{{{{\mathcal{T}}}}}}}}={{{{{{{{\mathcal{T}}}}}}}}}_{{{{{{{{\rm{s}}}}}}}}}+{{{{{{{{\mathcal{T}}}}}}}}}_{{{{{{{{\rm{p}}}}}}}}}$, where the serial part ${{{{{{{{\mathcal{T}}}}}}}}}_{{{{{{{{\rm{s}}}}}}}}}$ can be tuned by adjusting the time per operation *τ*_s_ and the parallel part ${{{{{{{{\mathcal{T}}}}}}}}}_{{{{{{{{\rm{p}}}}}}}}}$ is solely controlled by the number of processors *n*. We then optimize the combined energy cost function over ${{{{{{{{\mathcal{T}}}}}}}}}_{{{{{{{{\rm{p}}}}}}}}}$ using the fixed total time constraint and obtain the minimal energy cost for partial parallelization (Supplementary information, Sec. S1),

7 $$\frac{{W}_{{{{{{{{\rm{tot}}}}}}}}}^{{{{{{{{\rm{com}}}}}}}}}}{N}=kT\ln 2+\frac{a}{b{{{{{{{\mathcal{T}}}}}}}}}{\left(s\sqrt{bN}+\sqrt{p}\right)}^{2}.$$

Equation (7) interpolates between the purely serial implementation (3) (*p* = 0) and the completely parallelizable processor (4) (*s* = 0). In particular, the quadratic energetic advantage of the parallel computer is seen to be weakened when parallelization is reduced (Fig. 4a).

### Algorithmic parallelization overhead

Another important aspect of real-world algorithms, that ought to be accounted for, is that of parallelization overhead^27^. Parallelization indeed frequently requires the execution of additional overhead operations *N*_ove_. For example, an algorithm may need to distribute data to the parallel workers and then, at the end, another one collects data back from the workers. Usually, this overhead is a function of the number of processors used^27^. Because of the constant ${{{{{{{\mathcal{T}}}}}}}}$ assumption, the overhead either means that each processor needs to work faster in order to compensate for the overhead (Fig. 4b inset), or that one might use a stronger degree of parallelization $1 > {b}^{{\prime} } > b$, where $n(N)\propto {b}^{{\prime} }[N+{N}_{{{{{{{{\rm{ove}}}}}}}}}(n)]$ instead of *n*(*N*) = *bN*. We shall assume that the maximal available parallelization is already used and that overhead may only be compensated by adjusting the calculation speed *τ*_p_. We then obtain (Supplementary Information, Sec. S2),

8 $${\tau }_{{{{{{{{\rm{p}}}}}}}}}^{{{{{{{{\rm{ove}}}}}}}}}=\frac{{\tau }_{{{{{{{{\rm{p}}}}}}}}}}{1+{N}_{{{{{{{{\rm{ove}}}}}}}}}(n)/N}.$$

Owing to the time dependence of the dissipated work in Eq. (1), the energy cost of the parallel execution not only increases with the number of additional operations *N*_ove_ but also because of the necessary increase in processing speed. As a result, we obtain from Eq. (1) the total energetic cost for a general function *N*_ove_(*n*) (Supplementary information, Sec. S2),

9 $$\frac{{W}_{{{{{{{{\rm{tot}}}}}}}}}^{{{{{{{{\rm{ove}}}}}}}}}(N)}{N}=[N+{N}_{{{{{{{{\rm{ove}}}}}}}}}(n)]\frac{W({\tau }_{{{{{{{{\rm{p}}}}}}}}}^{{{{{{{{\rm{ove}}}}}}}}})}{N}=\left(1+\frac{{N}_{{{{{{{{\rm{ove}}}}}}}}}(n)}{N}\right) \\ \times \left[kT\ln 2+\frac{a}{b{{{{{{{\mathcal{T}}}}}}}}}\left(1+\frac{{N}_{{{{{{{{\rm{ove}}}}}}}}}(n)}{N}\right)\right].$$

The overhead thus causes the parallel computer to be less efficient than the serial computer for small problem sizes. This is because the Landauer part adds a fixed cost to Eq. (9), while the dissipative part will only be dominant for large *N*. However, the parallel implementation exhibits better scaling and becomes more energy efficient for larger problem sizes, even for large overhead, as illustrated, for concreteness and simplicity in Fig. 4b for a linear overhead, *N*_ove_(*n*) = *cn* (different overhead functions are analyzed in Supplementary Information, Sec. S2). This fundamental advantage of parallel computers holds as long as *N*_ove_(*n*) scales better than *n*^3/2^, or, equivalently, *N*^3/2^, since *n* ∝ *N* because of the fixed finite-time constraint. This scaling is modified to *n*^5/3^ for current electronic computers (Supplementary Information, Sec. S4).

### Leakage currents and provisioning

Non-ideal computers moreover have a base energy consumption caused by leakage currents^33–36^. For low-voltage, low-power circuits, the subthreshold current (also known as the weak inversion current) is the dominant component of the leakage current^34^. We assume that the devices are working in the typical regime where the subthreshold current scales linearly with the supply voltage *V* between drain and source^33–36^. However, we note that this assumption may not always be valid in the low-power regime^54–56^. We also assume that the subthreshold current is a linear function of the total processing time ${{{{{{{\mathcal{T}}}}}}}}$, as the processors can be put into deep sleep mode after and before running the computation to reduce leakage dissipation, and of the number of used processors *n*^33–36^. We further, suppose that the supply voltage is adapted to be proportional to the frequency *f*_op_. As a result, we have ${W}_{{{{{{{{\rm{lea}}}}}}}}}(N,{{{{{{{\mathcal{T}}}}}}}})=n\alpha {f}_{{{{{{{{\rm{op}}}}}}}}}{{{{{{{\mathcal{T}}}}}}}}=\alpha N$ for both serial and parallel realizations, where *α* is a circuit-specific constant^33^. The corresponding energetic cost per computation, ${W}_{{{{{{{{\rm{lea}}}}}}}}}(N,{{{{{{{\mathcal{T}}}}}}}})/N=\alpha$, is hence the same for both serial and parallel algorithms; it simply shifts Eqs. (3)–(4) by a constant amount (Supplementary Information, Sec. S4). We further note that one of the main issues that limit the use of nearly infinitely parallel computers in practice is the fact that all the additional CPUs need to be provisioned^33^. This comes with additional hardware and infrastructure (for example input/output, DRAM memory, data storage and networking equipment) that consumes energy even when the CPUs are in deep sleep mode. We may account for provisioning by adding a problem size independent constant *β* to the energetic cost, so that ${W}_{{{{{{{{\rm{pro}}}}}}}}}(N,{{{{{{{\mathcal{T}}}}}}}})/N=\beta /N$. The provisioning work creates overhead for the parallel computer, which makes it inefficient for small workloads. However, the effect of provisioning becomes largely irrelevant with increasing problem size, making a parallel computer still the ideal choice for large problems (Supplementary Information, Sec. S4).

### Reversible computing

For logically reversible computing, the quasistatic Landauer bound of $kT\ln 2$ may be reduced to zero^37,38^. As a consequence, only the finite-time contributions to the energetic cost (which are thermodynamically irreversible) remain in Eqs. (1)–(4). The difference in energy consumption between ideal reversible and irreversible serial computers becomes negligible at high clock frequencies, while the difference between ideal reversible and irreversible parallel computers is constant (Supplementary Information, Sec. S5).

## Discussion

We have used insights from nonequilibrium thermodynamics to develop a general framework to evaluate the fundamental energetic cost of finite-time parallelizable computing, including partial parallelization and parallelization overheads. Our main result is that the finite-time energy cost per operation of a fully parallel computer is independent of problem size and can realistically operate close to the quasistatic limit, in stark contrast to serial computers. This fundamental advantage of parallel computers holds as long as the overhead *N*_ove_(*n*) scales better than *n*^3/2^. For serial computers, the key limiting factor is the finite-time constant *a*. To enable a drastic increase in operation frequency without prohibitive energy consumption, *a* needs to be strongly reduced below its current value of *a* ≈ *kT*s in electronic computers.

On the other hand, the massive advantages of parallel- over serial computers may make it worthwhile to drastically rethink the design of computing hard- and software. From an energetic (which ultimately translates to performance) perspective, massively parallel computers with extremely high numbers of small cores and aggressive dynamic voltage and frequency scaling techniques could deliver orders of magnitude better performance per watt compared to CPUs with few large and complex cores—provided that well-parallelizable algorithms exist. Such algorithms could be quite wasteful in terms of the parallelization overhead and still deliver great performance advantages. Therefore, it seems worthwhile to invest significant research and development resources in the development of such CPUs and suitable software algorithms. Moreover, in light of this work, alternate computing technologies such as massively parallel DNA-^39–41^ or network-based^42,43^ biocomputers may already be closer to the optimal computers described here than current electronic computers: These computers use small DNA molecules or biomolecular motors as computing agents, which are cheap to mass-produce and can be added to the computation in amounts matching the problem size. Both approaches have been estimated to operate close to the Landauer limit per operation^43^. From the perspective of finite-time energy cost, immensely parallel computers, such as biological computers or computers with massively parallel architectures and many-core processors, thus offer a potentially large, fundamental advantage over today’s few-core electronic computer architectures.

## Supplementary information

Supplementary information

## Data Availability

The authors declare that the data used in this work is available within the paper, by using the corresponding equations with the parameters given in the graphs’ legend. The figures are created using python, and the notebooks can be found in the Github repository linked in the code availability statement.

## Code availability

Python code to re-create the figures can be found at https://github.com/thawn/fundamental_energy.
