# Supplementary material for: Fundamental energy cost of finite-time parallelizable computing
Source: Nat Commun. 2023 Jan 27;14:447. doi: 10.1038/s41467-023-36020-2 (PMC9883481; doi:10.1038/s41467-023-36020-2)
Supplement: Supplementary file 1 — Supplementary information [file 41467_2023_36020_MOESM1_ESM.pdf]

Supplementary Information: Fundamental energy cost of finite-time parallelizable computing

Michael Konopik, Till Korten, Eric Lutz, and Heiner Linke

S1. PARTIALLY PARALLELIZABLE ALGORITHMS

The energy cost of partially parallelizable problems (see Eq. (7) in the main text) is derived from three observations: (i) the problem can be split into a serial and a parallel part, $N = N_\mathrm{s} + N_\mathrm{p} = sN + pN$, (ii) corresponding to a total duration $\mathcal{T} = \mathcal{T}_\mathrm{s} + \mathcal{T}_\mathrm{p}$, where the respective serial and parallel time allocations, $\mathcal{T}_\mathrm{s}$ and $\mathcal{T}_\mathrm{p}$, can be freely chosen, except for the fixed total time constraint. Since we are interested in the optimal energy cost, it is important to further (iii) optimize the energy cost function over $\mathcal{T}_\mathrm{p}$, respecting the time constraint. The computation times for a single algorithmic operation, for either the serial or parallel computation, τ_s and τ_p are thus related by,

$$\mathcal{T}_\mathrm{s} = \tau_\mathrm{s} N_\mathrm{s} \quad \text{and} \quad \mathcal{T}_\mathrm{p} = \tau_\mathrm{p} N_\mathrm{p}/n(N_\mathrm{p}). \tag{S1}$$

The total energy cost follows from Eq. (1) as,

$$\begin{aligned} W_\mathrm{tot}^\mathrm{com} &= NkT\ln 2 + \frac{a}{\tau_\mathrm{s}} N_\mathrm{s} + \frac{a}{\tau_\mathrm{p}} N_\mathrm{p} \\ &= N\left[kT\ln 2 + \frac{s^2 N a}{\mathcal{T}_\mathrm{s}} + \frac{p^2 a N}{\mathcal{T}_\mathrm{p} n(N_\mathrm{p})}\right]. \end{aligned} \tag{S2}$$

Minimizing with respect to $\mathcal{T}_\mathrm{p}$ using (iii), we obtain for all $n(N_p)$,

$$\mathcal{T}_\mathrm{p} = \frac{p\mathcal{T}}{p + s\sqrt{n(N_p)}} \tag{S3}$$

Inserting this result into (S2), we then find,

$$W_\mathrm{tot}^\mathrm{com} = N\left(kT\ln 2 + \frac{aN\left[p + \sqrt{n(N_p)}s\right]^2}{n(N_p)\mathcal{T}}\right). \tag{S4}$$

Equation (7) eventually follows with $n(N_p) = bNp$.

S2. PARALLELIZATION OVERHEAD

There are many possible kinds of overhead. In the main text, we consider the simple linear form, which is linear in the number of processors and thus linear in the problem size for an ideal parallel computer. For simplicity, we assume that all available processors are used and therefore $n(N)$ is fixed. The overhead is accordingly taken into account by modulating the computation speed. With $N_\mathrm{g} = N + N_\mathrm{ove}(n)$ and $\tau_\mathrm{p} = \mathcal{T} n(N)/N$, the general time needed to solve the problem including the linear overhead is,

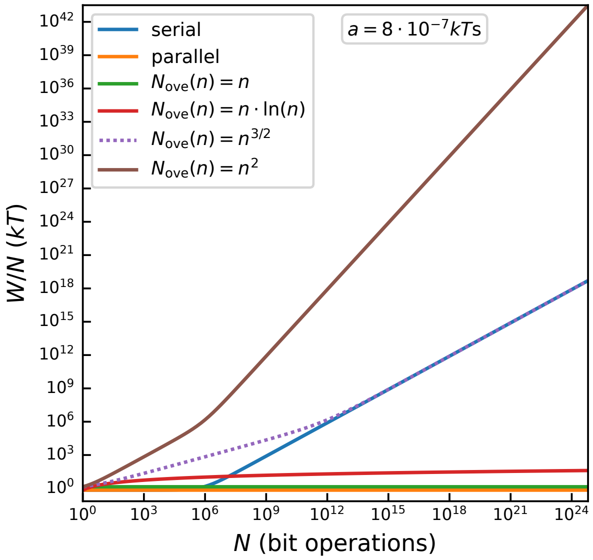

FIG. S1. Effects of different overhead behaviors. Energy cost per operation for a fully parallel algorithm with the given overhead function $N_\mathrm{ove}(n)$ in Eq. (S7). Parameters are $\mathcal{T} = 1$ s, $T = 1$ K, $b = 1$ and $a = 8 \cdot 10^{-7}$ kTs.

$$\mathcal{T}' = \tau_\mathrm{p}' \frac{N_\mathrm{g}}{n(N)} = \tau_\mathrm{p}' \left[\frac{N}{n(N)} + \frac{N_\mathrm{ove}(n)}{n(N)}\right]. \tag{S5}$$

The given time constraint is $\mathcal{T}' = \mathcal{T} = N\tau_\mathrm{p}/n(N)$ for the overhead-free case. We thus have,

$$\tau_\mathrm{p}' = \tau_\mathrm{p} \frac{1}{1 + N_\mathrm{ove}(n)/N}. \tag{S6}$$

For the overhead limited bound of finite time parallel computing, the total energy cost is finally,

$$W_\mathrm{tot}^\mathrm{ove} = N\left(1 + \frac{N_\mathrm{ove}(n)}{N}\right)\left[kT\ln 2 + \frac{a}{b\mathcal{T}}\left(1 + \frac{N_\mathrm{ove}(n)}{N}\right)\right]. \tag{S7}$$

Equation (S7) holds for any overhead function $N_\mathrm{ove}(n)$.

The precise form and scaling of parallel overhead depends on the nature of the problem being considered, as well as on its exact implementation. The overhead costs of serial and parallel implementations of various scientific programs have been investigated in Ref.[1]. Different scaling behaviors of the overhead function were evaluated for the following examples: (i) The Rabin-Miller test, which checks whether or not a number is a prime,

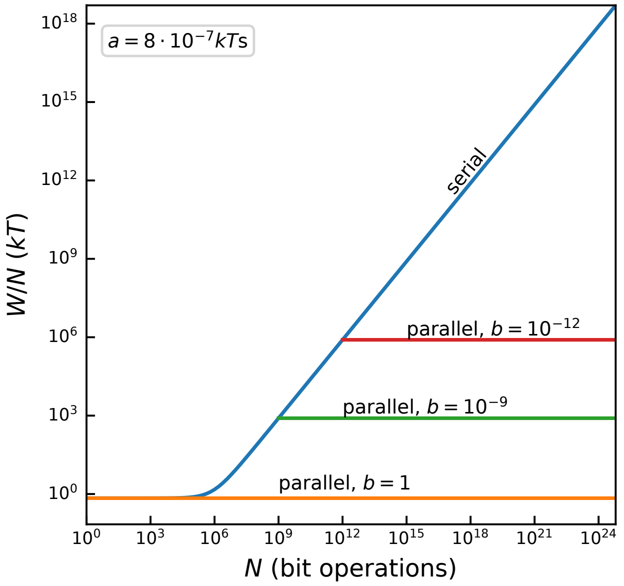

FIG. S2. Effect of different numbers of operations per parallel processor. Energy cost per operation for an ideal serial computer compared to ideal parallel computers with different numbers of processors. Parameters are $\mathcal{T} = 1$ s, $T = 1$ K, $b = 1, b = 0.001, b = 0.000001$ and $a = 8 \cdot 10^{-7}$ kTs.

corresponds to $N_{\text{ove}}(n) \propto n$ (Fig. S1, green line); (ii) A massive parallel simulation (using up to 250 thousand processors) of fluid flow and mass transport using the lattice-Boltzmann method, may be approximated by $N_{\text{ove}}(n) \propto n \ln(n)$ (Fig. S1, red line); (iii) The lower-upper (LU) decomposition of a $n \times n$ Pascal matrix, that scales as $N_{\text{ove}}(n) \propto n^2$ (Fig. S1, brown line). According to our model of parallel computing with overhead (S7), any overhead that behaves better than $N_{\text{ove}}(n) \propto n^{3/2}$ (Fig. S1 dotted purple line), results in an energetic benefit for parallel computing, compared to serial computing. Note, however, that modern day (non-ideal) electronic computers have a worse finite time scaling (see discussion in S4), meaning that for electronic computers, any overhead that behaves better than $N_{\text{ove}}(n) \propto n^{5/3}$, results in a benefit for parallel computing compared to serial computing.

S3. MORE OPERATIONS PER PARALLEL PROCESSOR

In the main text, we have defined the number of processors for a parallel computer as $n(N) = bN$ (with $b \in (0, 1]$) and set $b = 1$ throughout. The constant b thus determines the number of operations performed by each processor as $\nu = 1/b$. To illustrate the effect on the energy consumption, if we assume that one parallel processor performs 1, 1000 and 1000000 operations, we plot the energy consumption per operation for a parallel processor with $b = 1$, $b = 0.001$ and $b = 0.000001$, respectively, compared to a serial processor (Fig. S2). This increases the work per operation for the parallel processor by a constant factor, similar to a linear parallelization overhead (see main text Fig. 4b).

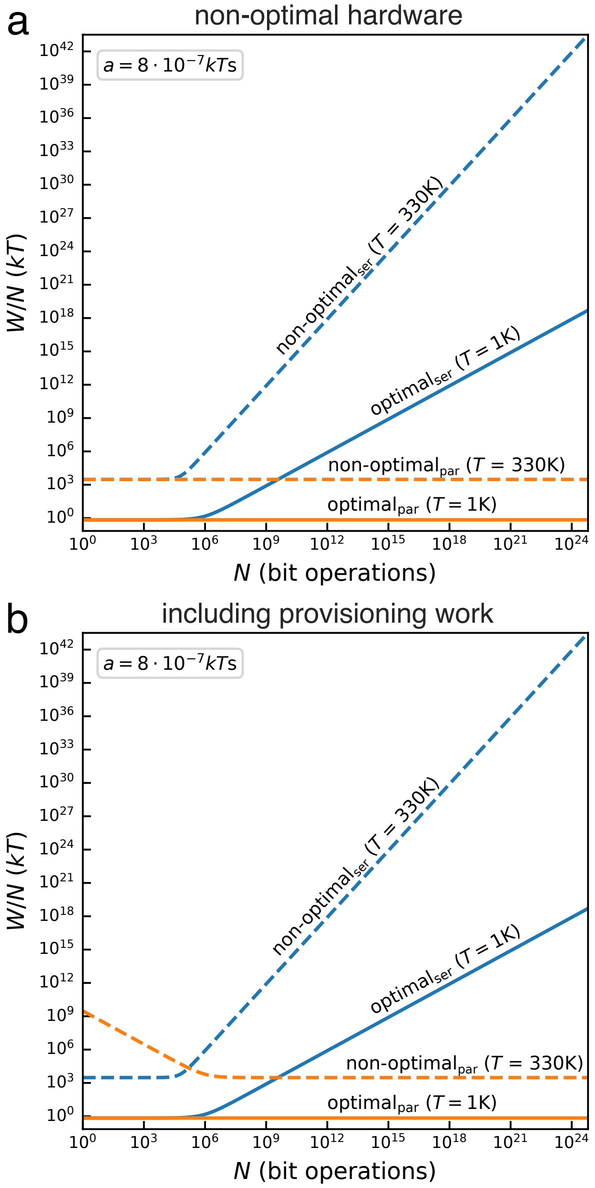

FIG. S3. Non-ideal computers. a) Energy cost per operation for optimal (straight lines) and non-ideal (dashed lines) serial (blue, Eq. (S8)) and parallel (orange, Eq. (S9)) computers. b) Same as a), except that the parallel computer includes provisioning work $W_{\text{pro}}(N, \mathcal{T})/N = \beta/N$. a, b) Parameters: $T_{\text{non-ideal}} = 330$ K (a typical temperature for a CPU under load), $T_{\text{optimal}} = 1$ K, $\mathcal{T} = 1$ s, $a = 8 \cdot 10^{-7}$ kTs, $b = 1$, $\alpha = 10^6$ kTs and $\beta = 10^{12}$ kTs.

S4. NON-IDEAL COMPUTER HARDWARE

So far, we have focused on optimal computer hardware, but real computers have additional costs. For one, we have considered the optimal $1/\mathcal{T}$ order, which implies a linear increase in energy dissipation with operation frequency $f_{\rm op}$. However, for today's electronic computers, the dynamic energy dissipation per computation grows approximately with $W_{\rm dyn}(V, f_{\rm op}) = \gamma V^2$ [5], where γ is a circuit-specific constant that we assume to be part of our constant a and V is the supply voltage. Further costs are incurred by leakage currents $W_{\rm lea} = \alpha V \mathcal{T}$ [5]. The overall energy dissipation for non-ideal hardware and N computations is hence $W_{\rm non}(V, f_{\rm op}) = W_{\rm dyn}(\mathrm{V}, f_{\rm op})N + W_{\rm lea}(V) + W_{\rm pro} = \gamma \mathrm{V}^2 N + \alpha V \mathcal{T} + \beta$. Because the supply voltage depends linearly on the frequency[5], we substitute V for $V = \mu f_{\rm op}$ and absorb γ, μ into a, giving us an overall energy dissipation of $W_{\rm non}(V, f_{\rm op}) = a f_{\rm op}^2 N + \alpha f_{\rm op} \mathcal{T}$. The energetic cost of the ideal serial computer then becomes

$$\frac{W_{\rm tot}^{\rm ser}(N, \mathcal{T})}{N} = kT \ln 2 + \frac{a}{\mathcal{T}^2} N^2 + \alpha \tag{S8}$$

For a parallel computer without overhead, each core has a constant operation frequency of $f_{\rm par} = 1/(b\mathcal{T})$. Therefore, the dynamic energy consumption has a in n constant effect. Further, the leakage currents dissipate additional energy for each core that is added, meaning that the energy consumption scales linearly with the number of cores $W_{\rm par}(n) = n W_{\rm lea}(f_{\rm op}) = n\alpha f_{\rm op} \mathcal{T}$. Also, provisioning work for parallel computer might be necessary $W_{\rm pro} = \beta$ [5]. As a result, the energetic cost of parallel computers becomes

$$\frac{W_{\rm tot}^{\rm par}(N, \mathcal{T})}{N} = kT \ln 2 + \frac{a}{(b\mathcal{T})^2} + \alpha + \frac{\beta}{N}. \tag{S9}$$

Interestingly, the cost incurred by the leakage currents are the same for both the serial (S8) and the parallel (S9) case, due to the fact, that the supply voltage dependence of these currents scales linearly with the operation frequency[5]. These costs are further also constant in total computation time $\mathcal{T}$, due to the fact, that a change in $\mathcal{T}$ is accompanied with a modification of the operation frequencies $f_{\rm op}$. The provisioning term β/N contributes to an increase in energetic cost for the parallel computer, which decreases with the problem size, becoming marginal for sufficiently large problems N. Without algorithmic overhead, the parallel computer has an even better scaling behavior than the serial computer, only limited by the size of the provisioning initially needed. Further interesting behavior can be found, when algorithmic overhead (S7) is considered with the non-ideal computer hardware. For general finite time scaling $W_{\rm dyn} = a/\tau^m$, one has the general form of the work cost

$$\frac{W_{\rm tot}^{\rm ser}}{N} = kT \ln 2 + \frac{a}{\mathcal{T}^m} N^m + \alpha \tag{S10}$$

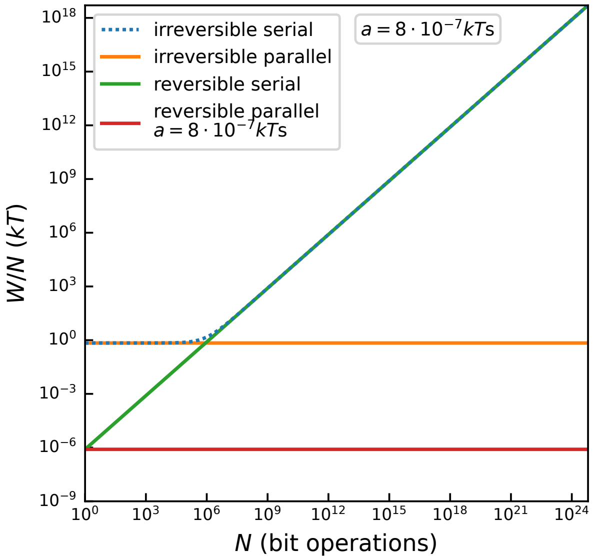

FIG. S4. Finite-time bound for reversible and irreversible ideal serial and parallel computers. Energy consumption per operation, W/N, for solving a fully parallelizable problem of size N by ideal serial, Eq. (3) (blue, green), and parallel, Eq. (4) (orange, red), computers operating irreversibly (blue, orange) or reversibly (green, red). The difference between ideal reversible and irreversible computers is a constant $kT \ln 2$. On the other hand, for ideal serial computers, the difference becomes negligible for anything but very small operation frequencies. Same parameters as in Fig. S1.

for the serial computer, and

$$\frac{W_{\rm tot}^{\rm par}}{N} = \left(1 + \frac{N_{\rm ove}(n)}{N}\right)\left[kT \ln 2 + a\left(\frac{1 + N_{\rm ove}(n)/N}{\tau_p}\right)^m\right] + \alpha\left(1 + \frac{N_{\rm ove}(n)}{N}\right) + \frac{\beta}{N} \tag{S11}$$

for the parallel computer, taking into account also the algorithmic overhead. The interplay between overhead and non-ideal properties of the computer results in a parallel work cost (S11) that now scales with the order m of the finite time dynamical work cost $W_{\rm dyn}$. From (S10), (S11), one is able to determine how large the algorithmic parallel overhead scaling can be, while still allowing for an energetically favorable parallel solution. For large N, the leakage currents and provisioning work becomes insignificant compared to the dynamical part in (S11), as the latter will always scale at least quadraticly with the overhead $N_{\rm ove}(n)/N$, and $m \geq 1$, while the former scale at maximum linearly.

The relevant m order behavior for the serial computer will then be $W_{\rm tot}^{\rm ser}/N \propto 1/\tau_s^m = N^m/\mathcal{T}^m$, while for the parallel computer one has for the highest m order behavior $W_{\rm tot}^{\rm par}/N \propto (N_{\rm ove}/N)^{m+1} \propto (N^k/N)^{m+1}$, for $N_{\rm ove} \propto n^k \propto N^k$ and $n = bN$. It thus follows, that the serial and parallel N scaling becomes identical, when $N^{2m+1} = N^{k\cdot(m+1)}$, or $k = (2m+1)/(m+1)$. If the overhead scales worse than $k \geq 2$, then no finite time

scaling m will satisfy this condition, and a parallel computer would scale energetically worse than the serial realization. For modern day electronic computers, the finite time scaling is given by $m = 2$, which would correspond to an energetically favorable scaling of the overhead when $k \leq 5/3$.

S5. REVERSIBLE COMPUTING

The Landauer limit of heat dissipation of $kT \ln 2$ per bit only applies for irreversible computing operations. For reversible computing this bound can in principle be brought down to zero[2–4]. In that case, the Landauer term $kT \ln 2$ may be dropped from Eqs. (3) and (4) (in the main text) and only the dissipative term related to finite-time effects remains. For ideal reversible serial computing, Eq. (3) thus becomes

$$\frac{W_{\rm rev}^{\rm ser}(N, \mathcal{T})}{N} = \frac{a}{\mathcal{T}} N = a f_{\rm op}. \tag{S12}$$

while for ideal reversible parallel computing Eq. (4) reads,

$$\frac{W_{\rm rev}^{\rm par}(N, \mathcal{T})}{N} = \frac{a}{b\mathcal{T}}. \tag{S13}$$

For an ideal serial computer, the difference between reversible (Fig. S4, green line) and irreversible (Fig. S4, blue dotted line) computing quickly becomes negligible as the number of operations per second increases. In this case, the increasing dissipative term dominates over the quasistatic limit — explaining why reversible computing has so far not gained any practical relevance. In contrast, a parallel computer, can take full advantage of the lower quasistatic limit of reversible computing (compare Fig. S4, red and orange lines). This indicates that future highly parallel computers may benefit from reversible circuit implementations.

SUPPLEMENTARY REFERENCES

[1] Lobachev, O., Guthe, M. & Loogen, R. Estimating parallel performance. *Journal of Parallel and Distributed Computing* **73**, 876–887 (2013).

[2] Bennett, C. H. Logical Reversibility of Computation. *IBM Journal of Research and Development* **17**, 525–532 (1973).

[3] Bennett, C. H. The thermodynamics of computation—a review. *International Journal of Theoretical Physics* **21**, 905–940 (1982).

[4] Bennett, C. H. Notes on Landauer's principle, reversible computation, and Maxwell's Demon. *Studies in History and Philosophy of Modern Physics* **34**, 501–510 (2003).

[5] Haj-Yahya, Jawad, Mendelson, Avi, Ben Asher, Yosi & Chattopadhyay, Anupam Power Management of Modern Processors In *Energy Efficient High Performance Processors: Recent Approaches for Designing Green High Performance Computing (Springer)*, 1–55 (2018).
